# Opioid doses required for pain management in lung cancer patients with different cholesterol levels: negative correlation between opioid doses and cholesterol levels

**DOI:** 10.1186/s12944-016-0212-9

**Published:** 2016-03-08

**Authors:** Zhenhua Huang, Lining Liang, Lingyu Li, Miao Xu, Xiang Li, Hao Sun, Songwei He, Lilong Lin, Yixin Zhang, Yancheng Song, Man Yang, Yuling Luo, Horace H. Loh, Ping-Yee Law, Dayong Zheng, Hui Zheng

**Affiliations:** Department of Oncology, Nanfang Hospital, Southern Medical University, #1838 Guangzhou Ave. N, Guangzhou, 510515 China; CAS Key Laboratory of Regenerative Biology, Guangdong Provincial Key Laboratory of Stem Cell and Regenerative Medicine, Guangzhou Institutes of Biomedicine and Health, Chinese Academy of Sciences, A-131, #190 Kaiyuan Ave, Guangzhou, 510530 China; Anhui University, Hefei, 230601 China; Sun Yat-sen University Cancer Center; State Key Laboratory of Oncology in South China; Collaborative Innovation Center for Cancer Medicine, #651 Dongfeng East Ave, Guangzhou, 510060 China; The third hospital, Southern Medical University, #183 Zhongshan Ave. E, Guangzhou, 510665 China; Department of Neurology, Nanfang Hospital, Southern Medical University, #1838 Guangzhou Ave. N, Guangzhou, 510515 China; Department of Pharmacology, University of Minnesota, Minneapolis, Minnesota, 6-120 Jackson Hall, 321 Church St. SE, Minneapolis, MN 55455 USA

**Keywords:** Cholesterol level, Opioid, Analgesia, Lung cancer patients

## Abstract

**Background:**

Pain management has been considered as significant contributor to broad quality-of-life improvement for cancer patients. Modulating serum cholesterol levels affects analgesia abilities of opioids, important pain killer for cancer patients, in mice system. Thus the correlation between opioids usages and cholesterol levels were investigated in human patients with lung cancer.

**Methods:**

Medical records of 282 patients were selected with following criteria, 1) signed inform consent, 2) full medical records on total serum cholesterol levels and opioid administration, 3) opioid-naïve, 4) not received/receiving cancer-related or cholesterol lowering treatment, 5) pain level at level 5–8. The patients were divided into different groups basing on their gender and cholesterol levels. Since different opioids, morphine, oxycodone, and fentanyl, were all administrated at fixed low dose initially and increased gradually only if pain was not controlled, the percentages of patients in each group who did not respond to the initial doses of opioids and required higher doses for pain management were determined and compared.

**Results:**

Patients with relative low cholesterol levels have larger percentage (11 out of 28 in female and 31 out of 71 in male) to not respond to the initial dose of opioids than those with high cholesterol levels (0 out of 258 in female and 8 out of 74 in male). Similar differences were obtained when patients with different opioids were analyzed separately. After converting the doses of different opioids to equivalent doses of oxycodone, significant correlation between opioid usages and cholesterol levels was also observed.

**Conclusions:**

Therefore, more attention should be taken to those cancer patients with low cholesterol levels because they may require higher doses of opioids as pain killer.

**Electronic supplementary material:**

The online version of this article (doi:10.1186/s12944-016-0212-9) contains supplementary material, which is available to authorized users.

## Background

As suggested in National Comprehensive Cancer Network (NCCN) guidelines, pain management contributes to broad quality-of-life improvement (www.nccn.org) [1, 2]. As pain killer for moderate to severe pain, opioids, like morphine, oxycodone and fentanyl approved by FDA, are widely used for pain management of cancer patients, particularly those with advanced diseases [3, 4]. Because of potential drug abuse, tolerance development, addiction and other side effects of opioids, opioid administration is under tight regulation to limit the usage and to avoid possible abuse. Opioids are normally administrated at relative low dose initially, and the dose is increased only if the pain is unchanged or increased during next pain level assessment. Therefore, the analgesia effects of opioids are important during pain management of cancer patients. Identifying the factors that affect opioid analgesia and understating the underlying mechanisms may provide a better protocol to treat cancer pain with opioids and improve patients’ quality-of-life.

In previous studies, cholesterol has already been identified as one of many factors that can affect functions of opioids [5, 6]. As a major mediator for morphine analgesia [7, 8], μ-opioid receptor (OPRM1 or MOR) locates in cholesterol-rich lipid raft micro-domain on cell membrane as some other G protein-coupled receptors (GPCRs) [9–11]. Cellular cholesterol regulates signal transduction of OPRM1 not only by supporting the entity of lipid raft micro-domain which subsequently maintains a specific membrane location for the interaction between OPRM1 and other signaling molecules, like G proteins and adenylyl cyclases, but also by stabilizing OPRM1 homodimerization and G protein coupling [5, 9]. Extracting cellular cholesterol or inhibiting cholesterol synthesis impairs the downstream signaling of OPRM1, like adenylyl cyclase inhibition, in HEK cell models and primary culture of rat neurons [6, 9, 12]. Actually, similar to the widely accepted understanding on GPCR and lipid raft [13, 14], the connection between cholesterol and opioid signaling has also be reported and confirmed by other laboratories. For example, reducing cholesterol level by methyl-β-cyclodextrin, a commonly used disruptor of lipid raft, impairs the signaling of δ-opioid receptor in neuronal cells [15], and cholesterol content is critical for δ-opioid receptor binding [16]. The contributions of cholesterol to OPRM1 signaling have also been described in rat caudate putamen [17], Chinese Hamster Ovary cells [18], and Human Embryonic Kidney Cells [19].

In addition, cholesterol level is important for opioid functions *in vivo*. By manipulating the lipid and cholesterol content in diet or administrating cholesterol lowering drug, simvastatin, the cholesterol levels in serum and brain of mice could be regulated successfully and significantly. Mice with high serum cholesterol levels require less morphine or fentanyl to achieve the similar analgesia effects than those with low serum cholesterol levels [6], suggesting analgesia abilities of opioids have a good correlation with cholesterol levels in mice. In addition, hypercholesterolaemic rabbits have significant increase in their responses to κ-opioid receptor agonist, which also suggests a correlation between high cholesterol level and enhanced opioid function *in vivo* [20]. Our previous studies also performed investigation on human subjects. When analyzing fentanyl usages for anesthesia before and during surgery, significant correlation was also identified between fentanyl usages and cholesterol levels in both male and female patients [6]. Thus, it is reasonable to hypothesize that patients with low cholesterol levels require higher doses of opioids for pain management.

In previous studies with human patients, fentanyl was used for surgical anesthesia and the correlation might not be applicable when opioids were used as pain killer [6]. Thus in current studies, an extensive study was carried out to identify potential correlation between cholesterol levels and opioid usages during cancer pain management. Clinical records of patients met certain criteria were selected out from about 9,000 patients with lung cancer.

## Results

### Clinical records of 282 patients are collected

The current studies were focused on patients with lung cancer, because lung cancer is the cancer with highest disease incidence and lethality rate in China [21, 22]. Improving pain management for these patients has significant social benefits.

There were about 9,000 patients with lung cancer received treatment during 2010 to 2014 in current database. The selection resulted in a pool of cancer patients enriched with those who were diagnosed as Phase III or Phase IV lung cancer during their first visit. Because of the unfortunate late diagnosis, these patients already suffered from moderate to severe pain (over level 5 as assessed by NCCN guideline) and required opioids as pain-killer, but they had not received surgery, radiation treatment, chemotherapy, or additional treatments for cancer-related syndrome. Totally 282 patients, 78 female and 204 male patients, were selected for further analysis.

### Collected records have normal distribution on cholesterol level

The clinical records of 282 patients were listed in Additional file 1: Table S1 because of the large file size. The heights and weights were used to calculate the body mass indexes (BMIs). Information on age, serum total cholesterol level, and opioid administration were also listed for further analysis.

**Fig. 1:**
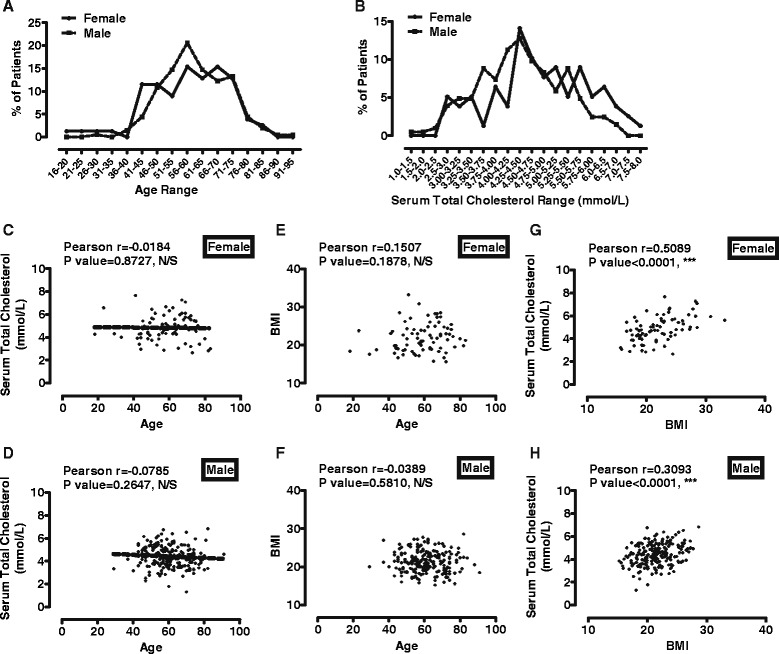
There is correlation between BMI and serum cholesterol level. **a** The distribution of ages of current 282 patients. The percentages of patients in each age range were plotted. **b** The distribution of serum total cholesterol levels of current 282 patients. The percentages of patients in each cholesterol range were plotted. **c**-**d** The correlation between serum cholesterol levels (y-axis) and ages (x-axis) was calculated with Pearson test in female (**a**) and male (**b**). **e**-**f** The correlation between BMIs (y-axis) and ages (x-axis) was calculated with Pearson test in female (**c**) and male (**d**). **g**-**h** The correlation between BMIs (y-axis) and serum cholesterol levels (x-axis) was calculated with Pearson test in female (**e**) and male (**f**)

After collecting all the necessary information, the distributions of patients on age and serum total cholesterol level were determined. As indicated in Fig. 1a, most female patients, 69 out 78 patients, were between 41 and 75 years old, and most male patients, 176 out of 204 patients were between 46 and 75 years old.

Most of patients have serum total cholesterol levels around 4.75 mmol/L, and female patients have slightly higher average cholesterol level than male (Fig. 1b). Such distribution is similar to that of Chinese population in 2012 [23]. In summary, 78 female patients have maximum cholesterol level at 7.66 mmol/L, minimal at 2.66 mmol/L, and average at 4.83 mmol/L. 204 male patients have maximum, minimal and average cholesterol level at 6.84, 1.31, and 4.41 mmol/L respectively. In addition, because of these different distributions of female and male patients on cholesterol level, they were analyzed separately.

### Serum total cholesterol levels correlate with BMIs

Before starting the actual analysis, correlations were determined between serum total cholesterol levels and ages, between BMIs and ages, and between serum total cholesterol levels and BMIs. By using Pearson test, no significant correlation was identified between serum total cholesterol levels and ages. *P* values were 0.8727 for female and 0.2647 for male (Fig. 1c-d). In addition, BMIs did not correlate significantly with ages (Fig. 1e-f). In female patients, Pearson’s rank correlation coefficient was 0.1507 (*P* = 0.1878, Gaussian approximation, *n* = 78). In male patients, Pearson’s rank correlation coefficient was −0.0389 (*P* = 0.5810, Gaussian approximation, *n* = 204).

However, consistent with previous report [6], there was significant correlation between BMIs and serum total cholesterol levels, suggesting patients with higher BMIs may have higher cholesterol levels (Fig. 1g-h). In female patients, Pearson’s rank correlation coefficient was 0.5089 (*P* < 0.0001, Gaussian approximation, *n* = 78). In male patients, Pearson’s rank correlation coefficient was −0.3093 (*P* < 0.0001, Gaussian approximation, *n* = 204).

### Low cholesterol patients have larger percentage to not respond to initial doses

78 female and 204 male patients were further divided into high cholesterol, medium cholesterol and low cholesterol groups basing on their serum total cholesterol levels. Each group included about one-third patients and the resulted medium cholesterol group had similar average cholesterol levels to those of original groups.

**Fig. 2:**
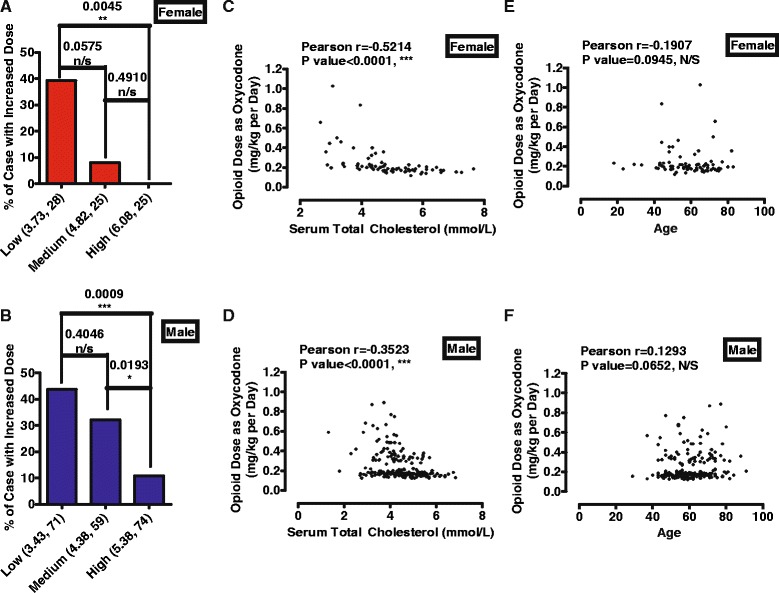
Low cholesterol patients are more likely to require higher doses of opioids. **a**-**b** Patients were classified into three groups, low, medium and high cholesterol groups, depending on serum total cholesterol levels. The average of serum total cholesterol levels and patients number were listed below x-axis. There were significant differences between high and low cholesterol groups in both female (**a**) and male (**b**). (**c**-**f**) The final doses of morphine and fentanyl used for patients were converted into oxycodone doses, and were plotted with serum cholesterol level (**c**-**d**) and age (**e**-**f**). Data for female and male were plotted in (**c** & **e**) and (**d** & **f**)

In female patients, 28 patients (<4.35 mmol/L) were classified into low cholesterol group with an averaged cholesterol level at 3.73 mmol/L, 25 patients (>4.35 and <5.30 mmol/L) into medium group with an average at 4.82 mmol/L, while the other 25 patients (>5.30 mmol/L) into high group with an average at 6.08 mmol/L. As indicated in Fig. 2a, 11 out of 28 (39.29%), 2 out of 25 (8.00%), and 0 out of 25 (0.00 %) patients in low, medium, and high cholesterol group did not respond to the initial doses of opioids. Fisher’s exact test suggested a significant difference between high and low cholesterol group (*P* value = 0.0045).

In male patients, 71 patients (<4.10 mmol/L) were classified into low cholesterol group with an averaged cholesterol level at 3.43 mmol/L, 59 patients (>4.10 and <4.70 mmol/L) into medium group with an average at 4.38 mmol/L, while the other 74 patients (>4.70 mmol/L) into high group with an average at 5.38 mmol/L. As indicated in Fig. 2b, 31 out of 71 (43.66%), 19 out of 59 (32.20%), and 8 out of 74 (10.81 %) patients in low, medium, and high cholesterol group did not respond to the initial doses of opioids. Fisher’s exact test suggested a significant difference between high and low cholesterol group (*P* value = 0.0009).

Since three kinds of opioids were used, each cholesterol group was further divided into three sub-groups according to which type of opioids they used. As summarized in Table 1, patients with low cholesterol level have higher probability to require higher opioid doses than those with high cholesterol levels. Significant differences were identified between high and low cholesterol group in male patients received fentanyl (*P* value = 0.0049, **) or oxycodone (*P* value = 0.0307, *) (Table 1). In addition, no significant difference was identified among different opioid sub-groups as determined by Fisher’s exact test.

**Table 1:** Percentages of patients not respond to initial dose with different opioid administration

78 female patients			
	Fentanyl	Morphine	Oxycodone
Low chol	5 (15)	1 (2)	5 (11)
Medium chol	0 (13)	0 (4)	2 (8)
High chol	0 (13)	0 (7)	0 (5)
Sum	5 (41)	1 (13)	7 (24)
204 male patients			
Low chol	11 (32)	2 (10)	18 (29)
Medium chol	0 (27)	1 (4)	10 (28)
High chol	1 (35)**	1 (8)	6 (31)*
Sum	20 (94)	4 (22)	34 (78)

Patients were classified into three groups, low, medium and high cholesterol groups, depending on their serum total cholesterol levels. Patients in each group were further divided into three sub-groups depending which opioid they were administrated. The numbers of overall patients and patients who did not respond to initial doses and required higher doses of opioids were provided. Fisher’s exact test was used to do statistical analysis between low cholesterol group with other two groups

Therefore, considering the fact that pain levels of patients were controlled at similar levels, from level 5 to 8, the observations suggested that patients with low cholesterol levels have higher possible to not respond to initial dose of opioids.

### Cholesterol level correlates with final opioid dose

Since no significant difference was identified between different opioids as determined by Fisher’s exact test (Table 1), the initial doses of these three opioids should be equivalent or close to equivalent to each other. Basing on the NCCN guidelines and previous reports on equivalent doses (Table 2) [6, 24–27], every 30 mg/day morphine sulfate in controlled-release tablets or 25 μg/h fentanyl in transdermal patch was calculated as 10 mg/day oxycodone hydrochloride in controlled-release tablet. The converted doses were plotted against the serum total cholesterol levels.

**Table 2:** The information for three opioids used for dose conversion

	Fentanyl	Morphine sulfate	Oxycodone hydrochloride
Formula	Transdermal patch	Controlled-release tablets	Controlled-release tablets
Minimal dose	25 μg/h per patch	10 mg per tablet	5 mg per tablet
Initial dose	25 μg/h	30 mg/day	10 mg/day
Initial dose converted to oxycodone	10 mg/day	10 mg/day	Not necessary
Initial dose equivalent to oxycodone	7.5 ~ 20 mg/day	10 ~ 20 mg/day	Not necessary

Basing on the NCCN guidelines and previous reports, doses of morphine and fentanyl in were converted to equivalent doses of oxycodone

As indicated in Fig. 2c-d, significant correlation between final opioid dose and serum total cholesterol level was found in both female and male patients. In female patients, Pearson’s rank correlation coefficient was −0.5214 (*P* < 0.0001, Gaussian approximation, *n* = 78). In male patients, Pearson’s rank correlation coefficient was −0.3523 (*P* < 0.0001, Gaussian approximation, *n* = 204).

In addition, no correlation was observed between final opioid dose and age (Fig. 2e-f). In female patients, Pearson’s rank correlation coefficient was −0.1907 (*P* = 0.0945, Gaussian approximation, *n* = 78). In male patients, Pearson’s rank correlation coefficient was 0.1293 (*P*-0.0652, Gaussian approximation, *n* = 204).

However, the correlation between cholesterol level and opioid dose might be attributed to the influences of opioids on cholesterol levels. The current studies were only focused on the initial doses of opioids, which were determined within the first week after opioid administration. Because of such a short time and relative low dose of initial opioid administration, cholesterol levels should not be affected significantly.

To further exclude this possibility, we selected and further analyzed the records of patients whose cholesterol levels were measured again during the first month after opioid administration. The reason why we limited such investigation within the first month was to prevent the influences from cancer progression.

In female patients, there were 16 patients had additional cholesterol measurement/s within the first month. For each of these 16 patients, all the measured cholesterol levels were normalized with the initial cholesterol level, and the largest difference in percentage was used for collective analysis. No significant change was identified on cholesterol levels were not significantly affected during first month after opioid administration (97 ± 9.1 %, *n* = 16, *P* = 0.1528, Additional file 1: Table S1). Similar phenomenon was also observed with 32 male patients (97 ± 12 %, *n* = 32, *P* = 0.7267, Additional file 1: Table S1). Thus opioid administration did not affect cholesterol level at least within our current paradigm.

## Discussion

As analyzed above, patients with low cholesterol have higher possibilities to require higher doses of opioids for pain management. Hence, we would like to suggest that pain level should be assessed more frequently or higher initial dose should be administrated for patients with low serum cholesterol levels in order to provide better pain management. Providing the patients with sufficient analgesic in a shorter time will reduce their suffering and improve their quality-of-life.

In addition, we also suggested that the cholesterol levels of patients should be considered when determining the initial dose of opioid administration to reduce the overall usage of opioids. For example, lower/higher initial dose of opioid should be administrated for patients with high/low serum cholesterol levels. During cancer progression, increasing doses of opioids are required for proper pain management not only because of the pain resulted from cancer enlargement and metastasis, but also because of the development of opioid tolerance. The side effects of opioid like sedation and respiratory depression prevent us to treat tolerance by simply and un-limitedly increasing opioid doses [28, 29]. Therefore, reducing the overall usage of opioids slows the development of tolerance down, increases the available time for opioids to control cancer pain and subsequent improves quality-of-life.

The mechanisms underlying the correlation between cholesterol level and opioid analgesia has been reported previously and mention in Introduction [5, 6]. Briefly, since opioids require proper signaling transduction of opioid receptor to function as analgesic, modulating opioid receptor is sufficient to affect the functions of opioids [30, 31]. In addition, cholesterol not only promotes the interaction between opioid receptor and downstream signaling factors as a main component of lipid raft micro-domain but also directly stabilize receptor homodimerization [5, 9]. Thus reducing cholesterol level impairs opioid function both in cells, in animal models [6, 9, 20], and there is a negative correlation between cholesterol level and opioid usage in current patients. Although the such correlation is weaker *in vivo* than *in vitro*, possibly because of the tighter regulation of cholesterol level in brain [32], it provides additional information for clinical application of opioids and agonists of other GPCRs. Since both the translocation of GPCRs into and out of lipid raft after agonist treatment have been reported [13, 33], it is reasonable to suggest the contributions of cholesterol to signaling transduction of other GPCRs, like Gonadotropin-releasing hormone receptors [34, 35], adrenergic receptor [36], cannabinoid receptor [37] and so on. Considering more than 40 % marketed drugs target GPCRs [38, 39], it is possible to identify additional correlation between cholesterol level and drug function, which will be critical for related disease treatment and may facilitate related research.

In current database, only 282 patients were selected from about 9,000 lung cancer patients for the following reason. Firstly, surgery, radiation treatment, chemotherapy, or additional treatments for cancer-related syndrome may affect pain level within a short time frame and introduce additional difficulties and un-accuracy to the determination of “final dose” of opioids. Thus we excluded these patients from further analyzed. Secondly, since some patients were initially treated in other hospitals, it was difficult to collect their full opioid administration information in current studies. Finally, the patients should suffer from at least moderate pain (level 5 as in NCCN guideline) and require pain management. These reasons were also why the 282 selected patients were all diagnosed as Phase III or Phase IV lung cancer during their first visit as cancer patient.

Since current patients were all diagnosed as Phase III or Phase IV lung cancer during their first visit as cancer patient, we could not determine the time interval between the emergence of cancer and first diagnosis, and subsequently had no access to the correlation between the duration of disease and the opioids. However, we suspected that there was an indirect connection between cancer progression and opioid efficacy *via* cholesterol levels. Weight loss during the late phase of cancer may also lead to the decrease in cholesterol level. Thus, during the cancer progression, the cholesterol level of patients decrease and subsequently requires higher dose of opioid. However, such correlation will not influences our conclusion, since current patients are in similar stage of cancer progression.

In current database, the patients were initially administrated with a fixed low dose of opioids. Thus patients who have lower BMIs or lighter body weights received higher dose of opioids initially as calculated as mg/kg. In addition, according to our observations, patients with lower cholesterol levels normally have lower BMIs and actually require higher dose of opioids. Therefore patients with lower cholesterol levels already received lower dose of opioids under current paradigm. Such fact of truth decreased the Pearson’s rank correlation coefficient of observed correlation at least partially, but also support our conclusion, low cholesterol patients require more opioids, since it is already applied in clinical practice though not purposely.

Basing on the NCCN guidelines and previous reports [6, 24–27], every 30 mg/day morphine sulfate in controlled-release tablets should be calculated as 10 ~ 20 mg/day oxycodone hydrochloride in controlled-release tablet. In order to make the initial doses of different opioids at similar levels, 30 mg/day morphine sulfate was calculated as 10 mg/day oxycodone hydrochloride. According the information provided in the instruction of current fentanyl patch, 25 μg/h fentanyl in transdermal patch could be calculated as 20 ~ 30 mg/day morphine. Thus we further calculated 25 μg/h fentanyl as 10 mg/day oxycodone hydrochloride in controlled-release tablet.

High serum cholesterol level has been suggested to be a high risk factor for cardiovascular diseases [40]. The cholesterol lowering drugs like statins have been used widely to prevents cardiovascular diseases in elder people [41, 42]. In addition, weight loss during the late phase of cancer may also lead to the decrease in cholesterol level. Thus cholesterol level of cancer patients may be lower than healthy controls [43–45], and should require higher dose of opioids as pain killer.

## Conclusions

1. Low cholesterol lung cancer patients are more likely to not respond to initial doses of opioids.
2. There is correlation between opioid usages and cholesterol levels.
3. Similar observations were obtained with three different types of opioids.

## Methods

### Clinical records retrieval

All current studies were based on the patients’ clinical records. The investigation abide by the Ethical Principles for Medical Research Involving Human Subjects outlined in the Declaration of Helsinki and was approved by the Institutional Review Board of Nanfang Hospital, Guangzhou, China. Written informed consents to share clinical records for medical and non-profit research were signed by corresponding patients or their representatives during treatment.

### Clinical records selection

Clinical records of about 9,000 lung cancer patients in current database of Department of Oncology, Nanfang Hospital, Guangzhou, China were used. Any record missing informed consents was removed from our selection. Personal information and information un-related to our studies were not included during data collection, transfer and analysis to protect privacy.

In current database, lung cancer patients with pain level over level 5 (assessed with NCCN guideline) were administrated with a relative low dose of opioids initially (initial dose), which was normally 10 mg/day oxycodone hydrochloride in controlled-release tablets, 30 mg/day morphine sulfate in controlled-release tablets, or 25 μg/h fentanyl in transdermal patch. Patients were re-visit on the next day or earlier if necessary to assess pain management. Only when the pain level was unchanged or increased, the doses of opioids were increased by additional one fold of initial dose. If a dose of opioid was able to control pain for two constitutive days, that dose was considered as final dose. Serum total cholesterol levels were required to be measured within one week from when final dose was determined.

Firstly, since opioid administration was recorded for all the patients, patients have serum total cholesterol levels measured within one week from when “final doses” of opioids were determined (normally during the first several days of opioid administration) were selected. Secondly, to reduce influences from the development of opioid tolerance, opioid-naïve patients were selected. Opioid-naïve patients were patients who have not received more than 60 mg/day morphine or other opioids at equivalent doses for three days in last one year. Patients who did not receive their initial dose at doses mentioned above were excluded in our analysis Thirdly, to avoid potential drug-drug interaction, we selected patients who required opioid as pain-killer (pain level over level 5), but had not received surgery, radiation treatment, chemotherapy, or additional treatments for cancer-related syndrome or other diseases. In addition, patients who were using cholesterol lowering drug like statins were also removed from our analysis [6]. Finally, to control the pain levels of the patients, we further selected the patients whose pain intensities were rated around level 5 to 8 with NCCN guideline.

### Pain assessment

Pain level information used in current studies was obtained from patients’ medical records. Normally, pain levels of patients were assessed once per day or when necessary following guideline provided by NCCN. Briefly, patients were asked about “current” pain, as well as “worst” pain, “usual” pain, and “least” pain in the past 24 h. Pain assessment was also helped by using “the Faces Pain Scale” as previously reported [46].

### Statistical analysis

Necessary data were collected from clinical records and listed in Additional file 1: Table S1 because the large quantity of the information. Statistical analyses were performed with GraphPad Prism 5.0. Pearson test was used to determine the correlation between two parameters and Pearson’s rank correlation coefficient and *P* values were provided. Linear regression was used determined the relation between ages and serum total cholesterol levels of patients, the slope and Y-intercept of the linear regression were provided. Fisher’s exact test was used to determine whether the patients with high cholesterol levels have higher possibility to require additional doses of opioids for pain management than those with low cholesterol levels. *P* values of Fisher’s exact test were provided. *,**, and *** were used to indicate a *P* value lower than 0.05, 0.01, and 0.001 respectively.

## Additional file

Additional file 1: A list of current patients’ information. The records of 282 patients were listed after removing primary information. ID was the reference number provided by the authors for easier accession. “Chol level” represented the serum cholesterol level measured and recorded. “Year” represented when the initial diagnosis of cancer was made. “Initial dose”, “stable dose” and “converted dose” represented the initial dose opioid administration, the final dose of opioids used for analysis and the dose when converted to equivalent oxycodone hydrochloride, respectively. The “increased” column, “1” or “0” were used to represented that “stable dose” was higher than “initial dose”. If the patient has one or multiple additional measurement of cholesterol during the first month after opioid administration, the cholesterol level with largest difference from initial cholesterol level was selected, recorded in “Chol level with largest difference” column, and normalized to the initial cholesterol level. (XLSX 43 kb)

## Abbreviations

BMI: body mass index
GPCR: G protein-coupled receptor
NCCN: National Comprehensive Cancer Network
OPRM1: μ-opioid receptor
